# Unraveling the mysteries of silver nanoparticles: synthesis, characterization, antimicrobial effects and uptake translocation in plant—a review

**DOI:** 10.1007/s00425-024-04439-6

**Published:** 2024-05-24

**Authors:** Ahmed Fares, Abdou Mahdy, Gamal Ahmed

**Affiliations:** 1 Plant Research Department, Nuclear Research Centre, Egyptian Atomic Energy Authority, Cairo, Egypt; 2 Plant Pathology Department, Faculty of Agriculture, Benha University, Benha, Egypt

**Keywords:** Silver nanoparticles, Synthesis, Antimicrobial effects, Translocation in plants

## Abstract

**Main conclusion:**

**The study thoroughly investigates nanosilver production, properties, and interactions, shedding light on its multifaceted applications. It underscores the importance of characterizing nanosilver for predicting its behavior in complex environments. Particularly, it highlights the agricultural and environmental ramifications of nanosilver uptake by plants**.

**Abstract:**

Nowadays, silver nanoparticles (AgNPs) are a very adaptable nanomaterial with many uses, particularly in antibacterial treatments and agricultural operations. Clarification of key elements of nanosilver, such as its synthesis and characterization procedures, antibacterial activity, and intricate interactions with plants, particularly those pertaining to uptake and translocation mechanisms, is the aim of this in-depth investigation. Nanosilver synthesis is a multifaceted process that includes a range of methodologies, including chemical, biological, and sustainable approaches that are also environmentally benign. This section provides a critical evaluation of these methods, considering their impacts on repeatability, scalability, and environmental impact. The physicochemical properties of nanosilver were determined by means of characterization procedures. This review highlights the significance of analytical approaches such as spectroscopy, microscopy, and other state-of the-art methods for fully characterizing nanosilver particles. Although grasp of these properties is necessary in order to predict the behavior and potential impacts of nanosilver in complex biological and environmental systems. The second half of this article delves into the intricate interactions that plants have with nanosilver, emphasizing the mechanisms of absorption and translocation. There are significant ramifications for agricultural and environmental problems from the uptake of nanosilver by plants and its subsequent passage through their tissues. In summary, by summarizing the state-of-the-art information in this field, this study offers a comprehensive overview of the production, characterization, antibacterial capabilities, and interactions of nanosilver with plants. This paper contributes to the ongoing conversation in nanotechnology.

**Graphical abstract:**

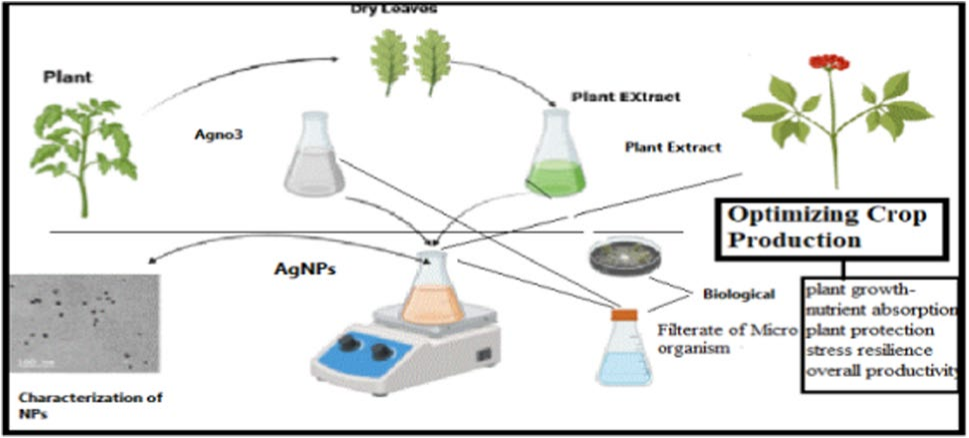

## Introduction

It has recently been suggested that nanomaterials might function as biostimulators to accelerate plant growth and multiplication (Thanagangelu et al. 2018). In addition, they have been linked to giving plants resilience to oxidative stress (Avestan et al. 2019); yet, they also provide difficulties for the micro- and macrobiota of organic soils. This disruption could alter, or perhaps completely destroy, the natural ecosystem of these microbes. Nanotechnology provides a competitive alternative to traditional chemical pesticides for the management of plant diseases (Janani et al. 2022). Examples of different nanoparticles are silver, copper, zinc, titanium, and carbon nanotubes (Anita et al. 2011). Nanoparticles are essential in agriculture because they promote crop growth and offer nutritional advantages. According to Elmer et al. (2016), innovative fertilizers for agriculture can use nanoparticles. Scientists have been very interested in the creation of noble metal nanoparticles because of their intriguing optical, electrical, magnetic, catalytic, and, most importantly, antibacterial capabilities. Thus, research efforts have been focused on developing environmentally acceptable production methods for noble metal nanoparticles. A material's ultrastructure can be examined at the nanoscale using transmission electron microscopy (TEM) and scanning electron microscopy (SEM), two powerful techniques. Through the use of an electron beam that is transmitted through the specimen, TEM technology enables high-resolution imaging of interior structures. SEM, on the other hand, collects information on surface morphology in detail by detecting electrons that are reflected from the sample’s surface. Moreover, a number of variables, including the use of surfactants like polyvinylpyrrolidone (PVP), which can stop nanoparticle aggregation and stabilize growth, can affect the production of silver nanoparticles (Sun et al. 2002). Furthermore, the synthesis technique selected can affect the nanoparticles' size, shape, and stability, with several techniques producing varied results (Dang et al. 2012). In conclusion, the production of silver nanoparticles involves a range of chemical and biological methods, each with unique advantages. Although chemical techniques are widely recognized and employed, biological technologies offer sustainable substitutes with a wide range of potential uses. Plant metabolites, surfactants, and reducing agent selection are some of the key factors that affect the properties of the generated silver nanoparticles.

## Nanoparticles approaches

**Fig. 1:**
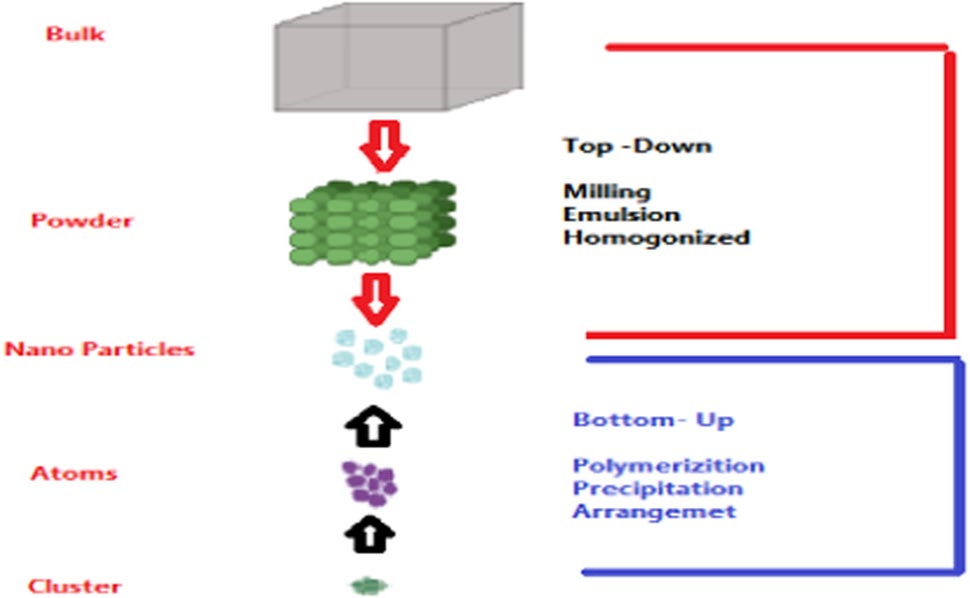
The diagram shows two approaches to nanoparticle synthesis (top-down and bottom-up)

Two approaches to nanoparticle synthesis (top-down and bottom-up) Fig. 1.

### Properties of silver nanoparticles

Because of their special qualities, a vast array of applications highly value silver nanoparticles. Because of their exceptional characteristics, which include size-dependent chemical, biological, and physical aspects, these nanoparticles have been the subject of much research (Hasim et al. 2020). The particle diameter of silver nanoparticles directly correlates with their thermodynamic characteristics, such as their melting point and molar heat of fusion (Tolaymat et al. 2010). Moreover, due to their ability to penetrate bacterial cell walls due to their nanoscale dimension and change cell membrane architecture, silver nanoparticles have been shown to have exceptional antibacterial characteristics (Yin et al. 2020a, b).The fact that silver nanoparticles are widely used in industrial applications such as electronics, photonics, and catalysis further justifies their selection for examination. According to Lekha et al. (2021), these nanoparticles’ special optical, electrical, and magnetic properties allow for their use in antimicrobial products, biosensors, textiles, cosmetics, composite fibers, and electronic components. Furthermore, it has been demonstrated that silver nanoparticles possess a range of diverse functionalities, such as antibacterial, medicinal, and property enhancement actions. As such, they can be effectively incorporated into a variety of materials, such as implantable devices, dental composites, and root canal fillers, in order to inhibit microbial growth and improve the material’s properties (Sakhamuri et al. 2023).The potential effects of silver nanoparticles on the environment and human health have also drawn criticism. These nanoparticles have a wide range of uses, but it has also been mentioned that they might be harmful to the environment and public health (Panyala et al. 2008). Understanding how to weigh the potential hazards and advantages of silver nanoparticles is crucial for their safe and effective use. All things considered, the special properties of silver nanoparticles—such as their size-dependent properties, antibacterial activity, and variety of functions—make them an appealing candidate for examination. It is crucial to consider the health and environmental consequences of using these nanoparticles in order to guarantee their suitability and sustainability.

### Green synthesis of silver nanoparticles

An environmentally responsible and sustainable method of producing nanoparticles is the green synthesis of silver. Numerous studies have looked into the potential of microorganisms and plants as biological sources for the manufacture of silver nanoparticles (Mustapha et al. 2022). Plant extracts, including those from *Syzygium alternifolium* and *Lonicera japonica*, have been shown to be useful in the environmentally friendly manufacture of silver nanoparticles (Balan et al. 2016; Yugandhar & Savithramma 2015).The potential applications of silver nanoparticles synthesized by environmentally friendly methods in disinfection and antimicrobial bandages have been highlighted due to their antibacterial capabilities (Willian 2023; Chopade & Kamble 2021). In addition, it has been demonstrated that adding honey and *Gymnema sylvestre* to the synthesis process may help produce silver nanoparticles with potential uses in medicine (Malik et al., 2020). The possibility of using green synthesis to create silver nanoparticles to cure cancer and fight microbial infections has also been studied (Ortiz 2017; Bhakat, 2012). Furthermore, it has been discovered that the biosynthesis of silver nanoparticles utilizing plant extracts has low toxicity and good thermal stability, making them appropriate for a variety of industries, including the production of drugs (Abdulkareem et al. 2021). With potential applications in materials with antibacterial properties, the green synthesis methodology offers a safer and more environmentally friendly method for producing silver nanoparticles (Ogunmodede et al. 2019; Ruttkay-Nedecky et al. 2019). In conclusion, a viable path for the sustainable synthesis of nanoparticles with a variety of uses in materials science, environmental protection, and medicine is provided by the green synthesis of silver nanoparticles utilizing plant extracts and other natural sources. To completely explore the potential of green-synthesized silver nanoparticles and their wider ramifications, more study in this area is imperative. Chemical, biological, and physical processes are the three primary processes utilized to produce nanoparticles. One method of creating nanoparticles is through physical processes such as evaporation and condensation in a tube furnace running at atmospheric pressure (Ku & Chung 2023).

### Synthesize of silver nanoparticles by plant extract, fungi, and bacteria

**Fig. 2:**
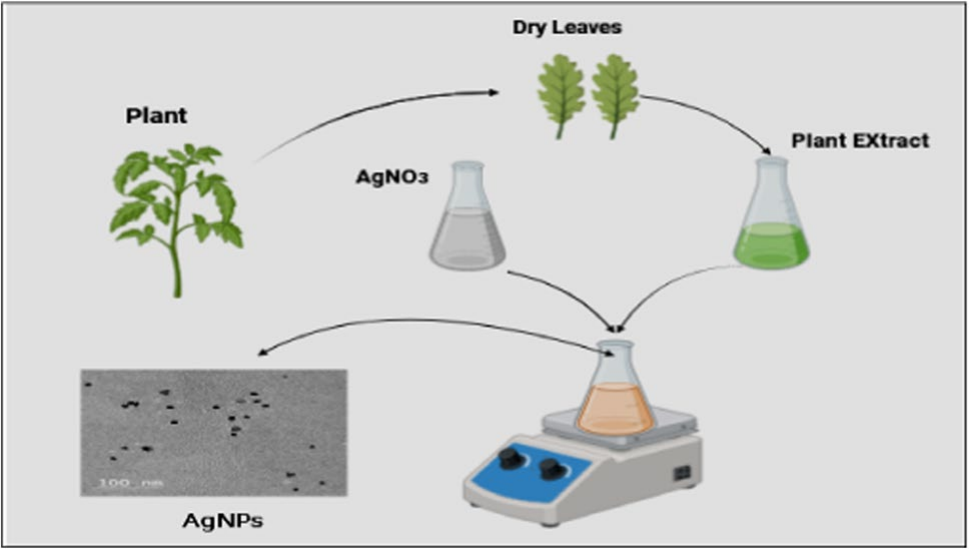
Production of silver nanoparticles from AgNO_3_ + plant extracts such as neem extract under stirring and use of TEM to characterize the size and shape of AgNPs

An important development in green nanotechnology is the use of fresh leaves, Fig. 2 seeds, fungal, and bacterial metabolites in the manufacturing process of silver nanoparticles (AgNPs) (Tariq., 2022). Conventional techniques for synthesizing AgNPs frequently rely on chemical agents, which can be resource and environmentally intensive. However, researchers have created environmentally benign methods for synthesizing nanoparticles by utilizing the inherent stabilizing and lowering abilities of microbial metabolites and plant extracts. Many bioactive substances found in fresh leaves and seeds, including phenolics, terpenoids, and flavonoids, function as capping and reducing agents to help turn silver ions into nanoparticles (Hasan et al. 2022). In a similar vein, biomolecules and enzymes generated by bacterial and fungal metabolites accelerate the reduction of silver ions, which results in the creation of AgNPs (Kulkarni et al. 2023). These biological sources have various benefits, such as reduced environmental impact, cost-effectiveness, and scalability. Furthermore, using plant and microbial extracts makes it possible to produce nanoparticles sustainably, which is in line with the ideas of green chemistry and nanotechnology. Therefore, the use of fresh leaves, seeds, fungi, and bacterial metabolites in the synthesis of AgNPs not only broadens the range of green synthesis techniques but also shows promise for the creation of novel, eco-friendly nanomaterials with a variety of uses in environmental remediation, agriculture, and medicine (Singh & Associates, 2023). Due to their accessibility and well-known medicinal qualities, neem leaves are frequently utilized in green synthesis. Moreover, it has been shown that the antibacterial activity of silver nanoparticles made by biosynthesis can be increased by neem leaf extract-synthesized silver nanoparticles (Fares et al. 2023b). It was discovered that after studying neem extract and its silver nanoparticles, the neem extract’s organic components worked well as a cap for the nanoparticles, promoting their stability and creation. During the process of creating silver nanoparticles, the aqueous extract of lemon leaves (Citrus lemon) is used as a reducing agent and a cage to retain the silver particles. According to Vankar and Shukla (2012), stable nanoparticles with a multi-shaped morphology and diameters of less than 100 nm of silver are produced when the extract from lemon leaves reduces the concentration of silver ions in the metal. These results show that plant extracts, especially those derived from neem and lemon leaves, are versatile and potent when manufactured in an environmentally responsible manner (Senthilkumar et al. 2018). However, because of their unique qualities, biologically produced nanoparticles, or NPs, are becoming more and more popular in the pharmaceutical sector. Sustainable and environmentally friendly processes are used to manufacture these NPs (Deepika et al., 2013). Plant extracts are excellent reducing and stabilizing agents for green NP production because they include a variety of secondary metabolites with strong reducing potential (Siddhant et al., 2017). A plant extract can function as a stabilizing agent and reducing agent during the NP synthesis process, minimizing the unintended agglomeration of colloids (Srikar et al. 2016). Unique chemical, biological, and physical characteristics are present in silver nanoparticles. They have great potential for nanobiotechnological applications and show notable catalytic and antibacterial activities (Muruzović et al. 2016). Metabolites of therapeutic significance, including secondary chemicals, biomolecules, and cofactors for enzymes, are present in the extracts of plants. These metabolites cause the decrease or stability of Ag + ions into AgNPs, increasing their therapeutic efficacy. Studies have demonstrated that, in comparison to the effects exhibited by solitary plant extracts, plant extract-coupled AgNPs demonstrate additive and synergistic effects on diverse biological processes, cancer cell lines, and microbial strains (Majeed et al. 2022).

### Efficacy of trisodium citrate and sodium borohydride (NaBH_4_) to create AgNPs

A substantial amount of a strong reducing agent, such as sodium borohydride (NaBH_4_), was used to create monodisperse and evenly sized silver colloids, allowing scientists to modify the structure and form of silver nanoparticles (AgNPs) (Pal et al. 2007). Biological methods, as opposed to chemical ones, provide greater control over the shape, size, and dispersion of nanoparticles through process optimization during synthesis. In addition, they used two effective and feasible ways to chemically reduce sodium borohydride (NaBH_4_) and trisodium citrate (TSC) to make silver nanoparticles (AgNPs). However, these nanoparticles differ in their ability to stop the growth of microbes because of factors affecting their toxicity capability. Improving the physical–chemical environment in the production process is an opportunity to raise the antibacterial activity of AgNPs. In addition, Quintero-Quiroz et al. (2019) chemically reduced AgNPs with sodium citrate (TSC) and NaBH4 to assess the antibacterial characteristics of AgNPs. Silver nanoparticles can be synthesized through various methods, including both chemical and biological approaches. Silver salts are reduced chemically by employing reducing agents including ascorbate, citrate, and NaBH4. These methods have been proven to work and are commonly used to produce silver nanoparticles (Najafi et al. 2021). On the other hand, biological techniques employ bacteria, microorganisms, or plant extracts to help reduce silver ions to nanoparticles (Mustapha et al. 2022). These biogenic synthesis techniques provide environmentally benign substitutes for conventional chemical processes (Gudikandula & Maringanti 2016).

### Evaluation of biological agents on the synthesis of nanoparticles

One benefit of using biological approaches is the availability of proteins, amino acids, and secondary metabolites during the creation of silver nanoparticles (AgNPs). One obvious benefit is the elimination of an additional step required to stop particle aggregation. In addition, the biological molecules used in the synthesis of AgNP do not cause pollution or harm to the environment. In these biological methods, fungi have been used, including *Fusarium oxysporum*. The biological synthesis of nanoparticles requires three essential components: a reducing agent, a solvent, and a non-toxic substance (Shankar et al., 2003). While using plant extracts to make nanoparticles can appear less cost-effective than using microbes, there are certain advantages to this approach. This method’s easy scalability makes it suitable for a variety of production volumes. Furthermore, it is suitable for the products’ intended application in the medical field and conforms with environmental safety laws (Gardea et al., 2003). Particle size and shape are crucial factors for a range of medical applications, and they can be controlled by biological processes employing bacterial proteins or plant extracts as reducing agents (Gurunathan et al. 2009). Due to their ability to produce a diverse range of chemicals, fungi are an invaluable resource for numerous purposes.

### Synthesize silver nanoparticles using physical methods

The production of silver nanoparticles using ball milling and laser ablation has garnered attention in recent study. Without requiring lengthy purifying processes, laser ablation has shown promise as a quick and effective method for producing silver nanoparticles in a variety of environments, including pure water (Mafuné et al. 2000). This technique shows off its potential for use in nanotechnology and healthcare by providing a simple and easy way to synthesize silver nanoparticles (D'Souza & Swarrup 2021). Conversely, ball milling has been acknowledged as a flexible technique for producing nanoparticles, particularly silver nanoparticles. Research has indicated that ball milling can be an effective method for synthesizing silver nanoparticles, resulting in consistent and stable particles that can be used in a range of applications (Venkatesh et al. 2022). In addition, ball milling has been employed in the preparation of silver nanoparticle–graphene oxide nanocomposites, demonstrating its adaptability in creating cutting-edge nanomaterials. The combination of laser ablation and ball milling techniques provides researchers with a comprehensive toolkit for the controlled synthesis of silver nanoparticles with tailored properties. Laser ablation enables the rapid and precise generation of silver nanoparticles, while ball milling offers a scalable and efficient method for producing nanoparticles with controlled size and morphology. By leveraging these physical methods, researchers can explore the structural, optical, and antimicrobial properties of silver nanoparticles, paving the way for their integration into diverse applications such as biosensors, antibacterial agents, and catalysis (Joy et al. 2022).The synergy between laser ablation and ball milling techniques not only facilitates the synthesis of silver nanoparticles but also opens avenues for the development of novel nanocomposites and functional materials with enhanced properties for various technological applications (Nguyen et al. 2023). Gamma radiation can be used to produce silver nanoparticles in a way that can be used to make them physically. One technique that uses the reduction of silver ions to generate nanoparticles is the creation of silver nanoparticles triggered by gamma radiation (Salleh et al. 2021). Using a radiation-induced process, this methodology forms silver nanoparticles by irradiating a solution containing silver ions (Lungulescu et al. 2019). The production process can be made simpler and more efficient using gamma radiation to synthesize silver nanoparticles (Ghobashy 2020). Gamma radiation serves as a catalyst for the reduction of silver ions in the physical synthesis of silver nanoparticles, which produces the nanoparticles in an appropriate media (Neimash et al. 2019). The radiation-induced chemical reaction of ionic silver reduction by gamma irradiation facilitates the immediate formation of silver nanoparticles (Neimash et al. 2019). Furthermore, it has been demonstrated that gamma radiation may effectively synthesize silver nanoparticles in a variety of matrices, including hydrogels, producing nanocomposites with distinctive characteristics (Ghobashy 2020). All things considered, the physical synthesis of silver nanoparticles via gamma radiation offers a viable method for the effective and regulated synthesis of nanoparticles. This technique provides a workable path for the synthesis of silver nanoparticles with potential uses in a variety of sectors, such as materials science and nanotechnology, by utilizing the concepts of radiation-induced reduction.

### Characterization of nanoparticles

Jiang et al. (2009) state that the many applications of nanoparticles depend on their unique characteristics, including size, shape, surface area, and dispersion. Nanoparticles need these qualities in order to perform effectively in a variety of settings. To evaluate the functional properties of the generated nanoparticles, characterization is essential, particularly for silver nanoparticles (AgNPs). Among the analytical techniques utilized for this include atomic force microscopy (DLS) and scanning electron microscopy (SEM), as well as Fourier transform infrared spectroscopy (FTIR), and transmission electron microscopy (TEM) (Zhang et al., 2016).

### UV–visible spectroscopy

One very helpful and reliable method for the initial characterization of generated nanoparticles is UV–Vis spectroscopy. It is also used to track the formation and stability of AgNP. It has been demonstrated that surface plasmon causes a distinctive peak to appear in a variety of metal nanoparticles with diameters ranging from 2 to 100 nm (Sastry et al. 1998). AgNPs’ distinct optical properties cause them to interact strongly with particular light wavelengths. One very effective, simple, quick, and sensitive technique for differentiating between different kinds of nanoparticles in colloidal solutions without the requirement for calibration is UV–Vis spectroscopy. It also takes the least amount of measuring time (Tomaszewska et al. 2013). UV–visible spectroscopy is the technique most frequently employed to characterize nanoparticles (Pal et al. 2007). Meticulous attention was given to the duration of mixing plant extract and silver nitrate solution to ensure optimal nanoparticle synthesis. Typically, the mixing process spanned from 30 min to 1 h, with variations based on specific protocols and experimental conditions. This careful control over mixing duration is crucial as it directly influences the size, shape, and stability of the synthesized nanoparticles. Adjustments were made to achieve desired nanoparticle characteristics and ensure consistency across experiments (Liaqat et al. 2022). In the UV–Vis spectroscopy section of the manuscript, the wavelength range used for monitoring AgNP synthesis was specified. UV–Vis spectra were typically recorded in the range of 300–800 nm, with the maximum absorbance peak corresponding to the surface plasmon resonance (SPR) of silver nanoparticles. This is the usual wavelength range used to evaluate the optical characteristics of AgNPs and characterize the manufacturing of nanoparticles. A fuller knowledge of the nanoparticle synthesis process was made possible by the analysis of the UV–Vis spectra, which validated the successful synthesis of silver nanoparticles and revealed information on their morphology and size distribution (Vasileva et al. 2021).

### Dynamic light scattering (DLS)

Dynamic light scattering (DLS) serves as a primary method for assessing particle sizes and size distributions in physiological or aqueous solutions, as emphasized by Murdock et al. (2008). This technique, as highlighted by Jiang et al. (2009), is particularly valuable for identifying particles suspended in a liquid based on their surface charge and size distribution. DLS quantifies the amount of light scattered from a laser as it passes through a colloid, utilizing Rayleigh scattering from suspended nanoparticles. The hydrodynamic size of the particles is then determined by analyzing the time-dependent change in scattered light intensity, as elucidated by Dieckmann et al. (2009). Monitoring the stability of mixtures over time was a critical aspect of the study. Various techniques, including UV–visible spectroscopy, dynamic light scattering (DLS), and zeta potential measurements, were employed to assess nanoparticle formation and stability during and after the mixing process. By monitoring variations in the surface charge, particle size distribution, and absorbance spectrum, researchers gained valuable insights into the kinetics of nanoparticle formation and stability under different conditions. This comprehensive analysis facilitated the optimization of the synthesis protocol and ensured reproducibility and stability of the synthesized nanoparticles (Rodriguez et al., 2023). DLS stands out as the only method that enables the nondestructive and simultaneous evaluation of numerous particles, despite certain sample-specific constraints, according to Kou et al. (2012). When conducting biological activity research and assessing the physical–chemical properties of nanomaterials produced for such purposes, radiation scattering techniques are essential. DLS plays a crucial role in this context, as it can examine the sub-micron to nanometer-sized distribution of tiny particles in suspension or solution, as highlighted by Lin et al. (2014). The application of DLS contributes significantly to our ability to understand and characterize nanomaterials, particularly in biological and physiological contexts, providing valuable insights into their behavior and potential implications.

### Fourier transform infrared spectroscopy (FTIR)

**Fig. 3:**
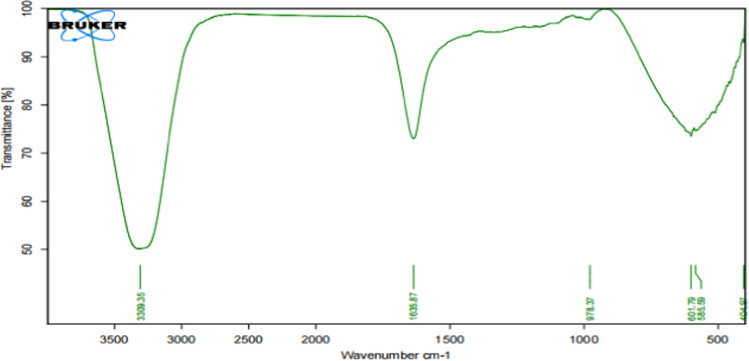
FTIR for AgNPs produced by biological agent

Fourier transform infrared spectroscopy (FTIR) stands out as a highly accurate and repeatable technique with an excellent signal-to-noise ratio, as highlighted by Zscherp and Barth (2001). This unique property enables the precise separation of tiny absorption bands associated with functionally active residues from the broader background absorption of the overall sample. FTIR spectroscopy plays a crucial role in evaluating the surface chemistry of nanoparticles due to its ability to detect surface chemical residues and organic functional groups attached to particle surfaces. The technique is based on the observation that subatomic particles within molecules undergo vibrations in relation to their original positions, and these molecular vibrations manifest as infrared (IR) modes. The appearance of these modes is linked to the fluctuation of the dipole moment. FTIR is indispensable for determining the presence of specific functional groups in a sample, as each functional group exhibits distinctive vibrational frequencies and sensitivity to the physiochemical environment, as noted by Chithrani et al. (2006).The technique has been utilized to measure parameters such as the decrease of Ag + ions and the protein molecules constituting the capping agent, as exemplified by the data presented in Fig. 3 from the study conducted by Fares et al. (2023a). The application of FTIR in examining the FTIR spectrum of silver nanoparticles enhances our understanding of their composition and paves the way for further advancements in nanomaterial research.

### Electron microscope and SEM characterization

Understanding of nanomaterials has been greatly enhanced by recent developments in high-resolution microscopy techniques, which have been made possible by advances in nanoscience and nanotechnology (Mallmann et al. 2015; Hahn et al., 2014). In particular, scanning electron microscopy (SEM) and transmission electron microscopy (TEM) have become essential tools for describing nanomaterials because they offer comprehensive details on the structure and form of individual particles. TEM, utilizing a more robust electron beam compared to SEM, achieves resolutions up to 1000 times higher, allowing for precise information on granularity and crystal structure at the atomic level (Eppler et al. 2000). Superior to SEM in terms of conducting analytical tests and spatial resolution, TEM is invaluable despite the drawback of requiring a tiny sample section under high vacuum conditions (Matsumura et al. 2003). One can use SEM and energy-dispersive X-ray spectroscopy (EDX) to investigate the morphology and chemical makeup of nanoparticles. Even though SEM cannot reveal internal structures, it is nonetheless useful for learning about particle cleanliness and aggregation. Nanoparticles smaller than 10 nm can be efficiently shaped using current high-resolution SEMs (Lemire et al. 2013). Surface electron microscopy (SEM) proves to be a powerful technique for comprehensively resolving particle sizes, size distributions, and nanomaterial shapes at both micro- and nanoscales. It facilitates the study of particle morphology and the generation of histograms from acquired images through specialized software or manual measurements (Tortella et al. 2021). The ability of TEM and SEM to magnify objects is one of their main differences. The higher magnification power that TEMs normally provide—up to 100,000 × —allows for the detailed viewing of tiny particles. SEM, on the other hand, is better suited for viewing bigger particles or surface characteristics because of its typically lower magnification power of about 50,000 × (Kanwal et al. 2021). Furthermore, there are differences in the imaging modes and resolution between TEM and SEM. While SEM is more frequently used for surface imaging and topography research, TEM is recognized for its high resolution, which makes it appropriate for in-depth structural analysis. For some investigations, such as grain boundary precipitates in materials like aluminum alloys, techniques like backscattered electron imaging in SEM can offer useful viewpoints (Ali et al. 2023). In conclusion, although TEM and SEM are both essential instruments in materials science, nanotechnology, and biological research, they are complementary methods that are selected in accordance with the particular goals of the study as well as the characteristics of the samples under investigation. These methods differ in their principles of operation, magnification capacities, sample preparation needs, and imaging modes.

### Impact of silver nanoparticles on microorganisms

Studies by Kumari et al. (2017) indicate that in order to enhance the antibacterial activity of silver nanoparticles (AgNPs), strategic control over their manufacturing, structural properties, and surface coating is necessary. The benefits that silver nanoparticles offer are numerous, as noted by Shahverdi et al. (2007). They are particularly efficient against a wide variety of germs because of their strong antibacterial properties. It is noteworthy that silver is both economically viable and has relatively minimal toxicity to humans at lower concentrations. Liu et al. (2010) have reported that several investigations have demonstrated evidence that the attachment of silver nanoparticles to bacterial surfaces may modify membrane function and raise the risk of bacterial toxicity. AgNPs have been recognized for their unique features, including their antibacterial, electrical, magnetic, and catalytic qualities. Agriculture makes extensive use of metal nanoparticles, including silver nanoparticles (Abdallah et al. 2019). According to recent research, nanoparticles may act as biostimulants to hasten plant growth and division (Saha et al., 2018).

Chemical reducing agents, which are used to create nanoparticles, are useful, but there are risks to the environment and public health that must be considered. The development of clean and ecologically sustainable technologies for the synthesis of silver nanoparticles is becoming more and more significant in the perspective of environmental sustainability. Silver nanoparticles are one often generated particle that inhibits the growth of fungus and bacteria (Khoshnamvand et al. 2020).

The growing use of nanosized silver particles has been facilitated by technological advancements that lower the cost of producing them (Young et al., 2009; Prasun et al., 2012). Silver nanoparticles are being used in many different fields, such as optics, electronics, mechanics, catalysis, energy research, medicine, and agricultural technology, because of their special physical and chemical properties (Elyasi et al. 2013; Sadeghi et al. 2013). According to Lamsal et al. (2011), AgNPs are also used as antibacterial treatments to manage plant diseases.

### Mode of action of silver nanoparticles in microorganisms

The way that silver ions alter the composition and form of microorganisms reveals their impact. An interesting discovery is that DNA molecules in microbial cells experience a transition to a condensed state upon the entry of silver ions, which stops them from reproducing and eventually leads to cell death. This phenomenon suggests a potential method by which silver ions modify DNA structure, hence blocking DNA replication. Research has demonstrated that silver and other heavy metals bind to thiol groups, disrupting protein activity and rendering the protein inactive (Feng et al. 2000). Ionic or nanoparticle silver's antifungal properties offer great hope for the treatment of spore-producing fungal plant diseases. Compared to synthetic fungicides, silver appears to be less dangerous for both humans and animals. When using chemical management techniques to combat plant fungal infections, it is important to take into account the strategic advantage of preventing the emergence of resistance. Various mechanisms of action that target different biological processes in microorganisms can be used to achieve this (Jo et al. 2009). Because of their antibacterial properties, silver nanoparticles, or AgNPs, have garnered interest as potential defense mechanisms for biological systems. Data clearly confirm the antibacterial properties of nanoparticles, although the exact mode of action is still up for debate. Many possible pathways for the harmful effects of silver nanoparticles on bacteria have been suggested by mechanistic investigations on the impacts of AgNPs. These include interactions via nanoparticles with sulfur in the cell wall and phosphorylated proteins, as well as adhesion to microbial surfaces that impacts membrane function (Liu et al., 2010).There are two main theories that account for Ag-NPs' antibacterial properties, although the precise mechanism is yet unknown. Ionic silver release and direct contact with the cell membrane are two of these possibilities. Several studies have shown that silver nanoparticles are potent antioxidants that can scavenge free radicals (Sivasankar et al. 2018; Rajoka et al. 2020). Theories that propose oxidative stress is caused by rupturing fungal cell membranes and generating reactive oxygen species (ROS) shed light on potential modes of action for AgNPs.

### Plant uptake and AgNPs translocation

According to studies, plants have the ability to absorb, translocate, and accumulate AgNPs from their surroundings (Tripathi et al. 2017). AgNPs are absorbed by plant roots more quickly than they dissolve in the soil, suggesting a quick absorption mechanism (Wu et al. 2021a, b). Furthermore, plants accumulate detectable levels of AgNPs through foliar absorption when the external dose is large, indicating that the predominant uptake route of AgNPs is concentration dependent (Dang et al. 2019). The significance of comprehending how plants alter and relocate AgNPs and Ag + ions have also been emphasized by research. Rice plants’ absorption, transformation, and translocation of AgNPs and Ag + ions have been shown through the use of dual stable isotope tracing techniques (Yang et al. 2018). In addition, plants can internalize silver through two different processes: either the roots dissolve AgNPs on the root surface, which allows the roots to internalize ionic species, or the roots directly absorb AgNPs and then release oxidized silver species within the root tissues (Yin et al. 2011). The absorption and transport of AgNPs in plants are significantly influenced by their size. Because of their larger surface area, smaller AgNPs are more cytotoxic and absorb more readily, which accelerates the release of Ag + ions into the plant system (Panzarini et al. 2018). Furthermore, the uptake of AgNPs can be influenced by their coating, as evidenced by the much increased Ag uptake seen in leaf cells exposed to certain coated AgNPs in comparison to other coatings (Štefanić et al. 2021). Comprehending the processes involved in the uptake and translocation of AgNPs in plants is crucial for determining the phytotoxicity of these particles as well as any risks that can result from their accumulation in the food chain and ecology. To fully understand the interactions between AgNPs and plants and their consequences for ecosystem health, more research in this field is necessary. Studies have shown how crucial it is to investigate how AgNPs affect plant gene expression because the discharge of AgNPs into the environment has sparked worries about how they may affect all living things, including plants. In addition, studies on AgNPs’ antibacterial qualities have demonstrated their potential uses in the fight against plant diseases (Yin et al. 2020a, b). In conclusion, complex processes driven by a variety of variables, including nanoparticle size, coating, and plant species, are involved in the uptake and translocation of AgNPs in plants. To properly understand the interactions between AgNPs and plants, their possible phytotoxic effects, and their uses in environmental management and agriculture, more research in this field is necessary. The absorption of silver nanoparticles (AgNPs) by plants has attracted a lot of interest because of its possible ecological effects, implications for agriculture, and environmental cleanup. Because of their special physicochemical characteristics, silver nanoparticles have potential uses in a number of industries, including agriculture, where they can be applied to improve nutrient intake, control disease, and encourage plant development. Research has shown that, depending on variables including nanoparticle size, surface coating, and plant species, AgNPs can enter plant tissues through a variety of routes, such as foliar spray and root uptake (Khan et al. 2023). After entering the plant, AgNPs might change, interacting with different parts of the cell and perhaps affecting the metabolism and physiology of the plant (Mikhailova., 2020). In addition, studies on the behavior and fate of AgNPs in plant tissues—including their translocation to other organs such as leaves, stems, and fruits—remain ongoing. In order to evaluate AgNPs’ potential as nanopesticides or nanofertilizers, assess their environmental impacts, and guarantee the safety of agricultural operations, it is imperative to comprehend the uptake mechanisms and subsequent effects of AgNPs in plants. Thus, more research into how plants and silver nanoparticles interact is necessary to maximize the useful uses of these particles while reducing any possible hazards to ecosystems and public health (Rehman et al. 2024).

### Effect of silver nanoparticles on plant growth and physicochemical properties

Despite worries regarding potential adverse effects on plant physiology, morphology, and growth during the absorption process, AgNPs may be beneficial for crop protection. As Khot et al. (2012) emphasized, more research is necessary to define possible hazards related to crops’ absorption of AgNPs, especially tomatoes, and the ensuing consequences for the environment and public health. AgNPs’ effects on tomato plants depend on a number of variables, such as the size, concentration, length of exposure, and physicochemical characteristics of the nanoparticles. Plant cells are mostly protected by enzymatic antioxidants from oxidative damage brought on by exposure to AgNPs. For example, *Wolffia globosa* exposed to 10 mg/L AgNPs experienced oxidative damage, which increased superoxide dismutase (SOD) activity by 2.52. AgNP-treated tomatoes showed similar results (Zou et al. 2016; Song et al. 2013). In addition, Geisler-Lee et al. (2014) have demonstrated that plant leaf biosynthesis represents an alternative pathway for AgNP absorption, supplementing the root pathway. AgNPs have demonstrated promise in crop protection, although worries have been raised about possible detrimental effects on plant physiology and growth during absorption (Spoială et al. 2020). Recent studies highlight the need for more investigation to comprehend the dangers connected to crops’ absorption of AgNP, especially tomatoes, and the ensuing consequences for the environment and public health (Yan and Chen 2020). The size, concentration, length of exposure, and physicochemical characteristics of the nanoparticles, among other parameters, influence how AgNPs affect plants (Ansari et al. 2023). Superoxide dismutase (SOD) and other enzymatic antioxidants are essential for shielding plant cells from oxidative damage brought on by exposure to AgNP (Rajput et al., 2020). Research has indicated that the Nicotiana tabacum plant may experience oxidative damage as a result of AgNP exposure, which would raise SOD activity (Biba et al. 2022). Furthermore, studies have shown that plant leaf biosynthesis can function as a supplemental mechanism to the root pathway for AgNP absorption (Xu et al. 2020). Even though there are worries that silver nanoparticles (AgNPs) may negatively impact plant growth, physiology, and morphology during the absorption process, recent studies have demonstrated the possible advantages of AgNPs in crop protection. There is a growing need for further investigation to understand the risks associated with AgNP absorption by crops, such as tomatoes, and the implications for both the environment and public health (Urnukhsaikhan et al. 2021). AgNPs have varying effects on plants, which can affect the function of enzymatic antioxidants in shielding Chlorella vulgaris from oxidative damage brought on by AgNP exposure. These effects include variations in nanoparticle size, concentration, exposure time, and physicochemical characteristics (Komazec et al., 2021). According to studies, plants may experience oxidative damage as a result of AgNP exposure, which may impact the activity of enzymes like superoxide dismutase (SOD). Furthermore, studies have shown that, in addition to the root system, AgNPs can also be absorbed by plants through other channels, such as plant leaf biosynthesis (Habeebet et al., 2022). AgNPs’ complicated and variable impacts on plant growth and health make a thorough study of how they interact with distinct plant species imperative.

### Exploring the dual impact of silver nanoparticles (AgNPs) in agriculture: enhancing growth vs. environmental and health risks

AgNPs, or silver nanoparticles, are being employed in agriculture more and more, which could be hazardous to the environment and biological systems. Research has demonstrated that AgNPs can build up in plants and impact their development and growth (An & Chen 2019). AgNPs can enter agricultural fields through biosolids from wastewater treatment plants, which can then introduce them into the environment. This can raise questions about the AgNPs’ potential effects on human health and food safety (Wang et al. 2013). Furthermore, it has been discovered that AgNPs have phytotoxic effects on plants, which affect early growth and germination (Yin et al. 2012). AgNPs’ ability to spread across food chains to creatures at higher trophic levels emphasizes the dangers they represent to the health of ecosystems (Wu et al. 2021a, b). AgNPs made with environmentally benign processes have potential for use in agriculture, but before they are widely used, further research is required to determine how harmful they are (Triveni & Nagaraju 2022). AgNPs’ antimicrobial qualities can be helpful in agriculture, but it is important to carefully examine any potential harmful impacts they may have on the environment and non-target organisms (Santos et al. 2021). Although the application of AgNPs in agriculture has demonstrated beneficial benefits on crop development, a complete assessment of the possible dangers associated with their long-term use is necessary (Mehmood 2018). All things considered, applying AgNPs to agriculture necessitates a balanced strategy that takes into account both the advantages of increased plant growth and productivity and the possible hazards to human health, the environment, and non-target creatures. To completely comprehend the ramifications of AgNP use in agriculture and to create plans to counteract any negative consequences, more research is necessary.

### Future perspective

Optimizing synthesis: future research should focus on optimizing nanosilver synthesis methods, considering both efficiency and environmental impact, to enhance their applicability across various fields. Advanced characterization techniques: continued advancements in characterization techniques will contribute to a more nuanced understanding of nanosilver properties, aiding in the development of safer and more effective applications. Tailored antimicrobial applications: investigating specific applications of nanosilver in combating emerging microbial challenges, including evolving antibiotic-resistant strains, can lead to tailored and targeted antimicrobial solutions. Environmental impact assessment: future studies should deepen our understanding of the environmental impact of nanosilver, particularly its long-term effects on soil, water, and ecosystems, ensuring sustainable agricultural practices. Plant–nanosilver interactions: further exploration of plant–nanosilver interactions, including cellular responses and potential benefits or risks to plant growth, will contribute to informed decisions regarding the use of nanosilver in agriculture. Regulatory considerations: as nanosilver applications expand, regulatory frameworks should be developed or refined to ensure responsible and safe use, balancing innovation with environmental and human health considerations.

## Conclusion

In conclusion, the exploration of nanosilver presented in this review underscores its extraordinary adaptability and range of uses, especially in the fields of agricultural practices and antimicrobial therapies. The synthesis methodologies discussed, ranging from traditional chemical approaches to sustainable and eco-friendly strategies, exemplify the dynamic landscape of nanomaterial production. The pivotal role of characterization techniques in unraveling the intricate physicochemical attributes of nanosilver cannot be overstated. Spectroscopy, microscopy, and other advanced analytical methods highlighted in this review serve as indispensable tools in advancing our understanding of this nanomaterial. The extensive investigation into nanosilver’s antimicrobial properties, specifically its interaction with bacterial cell membranes and its potential for addressing antibiotic-resistant strains, reflects a promising avenue for future therapeutic applications. The significant emphasis on its impact on plants, from uptake mechanisms to translocation processes, elucidates the complex interplay between nanosilver and the agricultural ecosystem.

## Data Availability

All data in this manuscript are available upon request.
